# Core Competencies of Truck Drivers Responding to Emergencies during Transportation of Hazardous Materials

**DOI:** 10.5696/2156-9614-10.27.200909

**Published:** 2020-08-25

**Authors:** Adnan Fazal Manzoor

**Affiliations:** COMSATS University Islamabad, Attock Campus, Pakistan

**Keywords:** HAZMAT Handling, Emergency Response, Competencies, Training, Risk

## Abstract

**Background.:**

Hazardous material (HAZMAT) transportation drivers are responsible for safe delivery of consignments and face multiple challenges carrying out their duties. Drivers are also the first to respond to emergencies and accidents.

**Objectives.:**

The purpose of the present study was to identify the essential competencies needed by HAZMAT transportation drivers to deal with emergencies.

**Methods.:**

Three rounds of focus groups were conducted using expert panels comprised of HAZMAT specialists, health, safety and emergency representatives, security experts and transportation advisors from June to July 2019. The panel discussed competencies, gathered from a literature review, for emergency responders.

**Results.:**

The panel identified six (6) core and 23 sub-competencies of HAZMAT drivers. This is the first study in low- and middle-income countries (LMIC) to identify core competencies of HAZMAT truck drivers.

**Conclusions.:**

The integration of these competencies into a development and training program for drivers will better enable drivers to handle emergencies in an efficient and effective manner.

**Participant Consent.:**

Obtained

**Ethics Approval.:**

The Graduate Advisory Committee of Comsats University approved study protocols.

**Participant Consent.:**

Obtained

**Competing Interests.:**

The authors declare no competing financial interests.

## Introduction

Due to its strategic location, Pakistan is an important key player in international energy routes and is continuously investing its resources in the establishment of industrial zones and sectors. Towards this end, the Gwadar Port and China– Pakistan Economic Corridor (CPEC) are critical to international trade.^1^ Once completed; the Belt and Road Initiative project would be a massive industrial advancement, covering approximately two-thirds of the land mass in 65 countries. The major important segments in this project are the Bangladesh-China-India-Myanmar Economic Corridor, China-Mongolia-Russia Economic Corridor, China-Indochina Peninsula Economic Corridor, China-Central and Asia-West Asia Economic Corridor and the New Eurasian Land Bridge.^2^ The land routes consist of high speed railways, highways, ports and industrial zones with a total length of 60,000 km. The result will be extensive trade and transportation of goods across the globe. The CPEC will offer a shortened route of 3,000 km compared to the existing route of 12,000 km for the oil market between the Middle East and Chinese border. The major transportation item on the CPEC is oil from the Middle Eastern Peninsula to Chinese territory for energy production.

The transportation of oil involves travel through pipelines, rail and road tankers. The transportation of liquid flammable material is labeled as Class 3 hazardous material (HAZMAT).The accidental release of these materials may have catastrophic effects for surrounding populations; the most significant effects are mortality, morbidity, environmental damage, and financial loss. Acute and immediate health effects with physical symptoms can occur during clean-up work or residential exposure and differ according to the type of material involved, for example ammonia versus hydrogen sulphide, etc. With increased transportation and handling of Class 3 HAZMAT come increasing challenges to the safe performance of these operations, as the incidence of Class 3 HAZMAT accidents is very high compared to all classes of HAZMAT.^3^ Analysis of previous accidents reveals that two-thirds of HAZMAT accidents occur on roads.^4^ In the United States, about 54% of HAZMAT is transported via trucks and the majority is Class 3 HAZMAT (78%).^5^ Presently, 80% of Chinese import oil is shipped through the Strait of Malacca and the current shipping time is more than 45 days. Following the commissioning of the CPEC, bulk quantities will be shipped through the CPEC and shipping time will be shortened to 10 days.

Extensive studies have been undertaken and various models proposed to address the issue of safe transportation of HAZMAT.^6^,^7^ The major underlying factors which significantly contribute to HAZMAT accidents and spillage have been identified as lack of awareness, non-adherence to safety rules, poor accident reporting mechanisms and poor licensing procedures.^8^,^9^ Human factors such as driver skill and awareness are major factors in these accidents, followed by vehicles, equipment and HAZMAT packaging.^10^ Inexperienced drivers in the HAZMAT transportation industry are a significant problem, leading to road accidents and improper emergency responses due to driver error.^10^,^11^ Driver performance is usually dependent on age, physical condition, expertise and experience.^12^ Fatigue is also considered to be a contributing factor in accidents.^13^ A previous study in Africa outlined some important measures to mitigate and prevent these incidents, including provision of standard driving hours with compulsory breaks, availability of intermediate stations for rest and food, and the availability of a HAZMAT emergency response team to initiate a timely response in case of accidents.^14^ Unfortunately, in Pakistan, working conditions for drivers are not conducive to occupational safety. For instance, drivers do not have proper rest cycles. They often experience extended periods of driving with no replacement/standby driver, which is a major factor in driver fatigue. Often the driving duties are shared with an assistant who is new to driving and usually gets training while on the road, an illegal and unsafe practice.

**Abbreviations**
CPEC: China–Pakistan Economic Corridor
HAZMAT: Hazardous Material
PPE: Personal Protection Equipment

The environmental consequences of HAZMAT accidents include air pollution, followed by water, soil and biological degradation.^11^ In proposed HAZMAT models, population exposure to HAZMAT accidents is considered to be less significant compared to environmental pollution.^14^ However, there have been disasters involving HAZMAT accidents which have resulted in the deaths of hundreds of people.

A tragic incident resulting in the death of more than 200 people in Ahmedpur East, Pakistan has called into question the effectiveness of the HAZMAT transportation safety system and follow-up management of HAZMAT incidents.^15^ Pakistan does not have a national level survey regarding HAZMAT accidents; therefore the present study uses anecdotal evidence regarding the poor state of HAZMAT transportation. Many incidents have been reported in the media, both electronic and print, highlighting the issue. For example, 33 people died in a collision between an oil tanker and a bus in 2011 in Jamshoro, Pakistan; in two separate incidents, 40,000 L of oil was spilled as tankers overturned in 2018, in both cases the cause of accident was attributed to the drivers.^16^–^18^ In another incident, more than sixty people died through a combination of impact, and fire sparked by the HAZMAT, attributed to negligence of the HAZMAT truck driver.^19^ These incidents shed light on the poor state of HAZMAT transportation in Pakistan; in the majority of the cases the driver is blamed. In addition, the literature review highlighted several HAZMAT incidents and accidents which resulted in loss of life, property damage and environmental contamination.^3^,^11^ Similar needs in terms of driving safety, communication and handling of hazardous materials are anticipated for other countries in the region, for example Vietnam, Indonesia, India and Sri Lanka.^5^

To prevent on-road HAZMAT accidents and mitigate the effects of HAZMAT release, the role of truck drivers and emergency responders is extremely critical. The role of emergency first responders is normally filled bypolice, firefighters, emergency medical nurses and HAZMAT specialists.^20^ Extensive role-specific training programs are essential to the development of emergency responders to mitigate the impact of HAZMAT occurrence.^20^ The degree of HAZMAT preparedness varies among various professional groups, however previous studies revealed that HAZMAT teams have a better response compared to firefighters and police.^21^,^22^ In the United States, police cadets are trained on HAZMAT-related issues in the police academy, and law enforcement personnel are trained to handled HAZMAT incidents.^23^,^24^ The necessary competencies identified for firefighters include the ability to conduct risk assessments, perform cardiopulmonary resuscitation and handle relevant equipment. Some emergency medical staff are been thoroughly trained in handling HAZMAT related incidents, especially risk assessment, hazard identification, toxicological effects of exposure to HAZMAT and treatment.^25^ The important competencies required in the response phase of emergencies are listed in Table 1.^26^,^27^

**Table 1:** Competencies of Incident Management Teams

**Competencies identified by Hayes *et al*.^26^**	**Competencies identified by Van Wart *et al*.^21^**
Interpersonal skills	Self confidence
Leadership	Willingness to assume responsibility
Decision-making ability	Motivating
Situation awareness	Articulating vision and mission
Communication skills	Resilience
Technical expertise	Communications skills
Management skills	Decision making
Calm and levelheadedness	Analytic skills
Problem solving skills	Decisiveness
Disciplined	Flexibility
Flexible	Delegating Operations planning Team building Networking Social skills

The fourth and most important professional group is the HAZMAT response team. A HAZMAT response team is a group of hazardous material experts who specialize in detecting, containing, and removing any release of HAZMATs in order to control an accident or incident.^28^,^29^ Researchers recommend further studies to determine the role and responsibilities of response teams.^30^ The positioning and timing of response teams at the incident site is a key factor in effective responses.^31^ However, many studies have overlooked the very first response initiated by the HAZMAT driver and crew members. The role of HAZMAT drivers and crew members is critical to effective mitigation measures against HAZMAT accidents. Due to the lack of time and resources in such cases, drivers must respond in multiple capacities, i.e. firefighter, HAZMAT responder, etc. This situation demands that drivers develop a set of core competencies to counter possible adverse effects of HAZMAT incidents. Drivers and crew members should possess some of the key competencies of fire fighters, HAZMAT responders, police and emergency responders outlined above. Competency is defined as possession of particular skills to execute tasks in a complex and challenging environment. The aim of the present study was to explore and identify the core competencies of drivers responding to emergencies during the transportation of hazardous materials.

## Methods

A qualitative and exploratory study was conducted from June to July 2019. An expert panel was convened comprised of two HAZMAT drivers, two health and safety experts, two firefighters, a rescue 1122 representative, an emergency nurse and a law enforcement officer. The Graduate Advisory Committee of Comsats University approved study protocols. Informed consent was obtained from participants prior to study conduct. Three meetings were convened from June to July 2019 at a local university in Attock, Pakistan. Data were collected through the focus group technique. To increase credibility, methodology triangulation was used through the focus group technique, literature review and a HAZMAT response video. In the first phase, the focus group was briefed on the objective of the study and working mechanism followed by extensive literature review using search engines, Google Scholar, Directory of Open Access Journals and Pub Med. Information was shared on the theme of HAZMAT transportation accidents and competencies, with reference to known HAZMAT incidents in Pakistan, especially the oil tanker accident at Ahmedpur East, in which an oil tanker was overturned on the road. Local residents gathered at the site of the accident in order to gather oil from the spill, when the tanker ignited, exploded, and resulted in the deaths of hundreds of people.^32^

In the second meeting, a response video on HAZMAT transportation accidents was presented to the group members to provide information on the theme. Afterwards, each member gave a presentation on five competencies along with their justifications. The recommended competencies were compiled.

In the third meeting, the group members were divided into subunits comprised of two members in each unit and the compiled list of competencies was distributed among the subunits for discussion and alteration, recommendation, and approval of competencies. After thorough deliberation and discussion, only the approved competencies and sub-competencies from all subunits were included in the final list of competencies. A thematic analysis of the collected data (the list of compiled competencies and recorded observations) was performed to obtain results for core and sub-competencies.

## Results

A total of six core and 23 sub-competencies were identified as a result of the thematic analysis. The detailed description of core and sub-competencies is provided in Table 2.

**Table 2:** Core and Sub-Competencies of HAZMAT Drivers Responding to Emergency Situations

**Core competencies**	**Sub-competencies description**
Awareness and knowledge of **assigned duties** as HAZMAT transport driver	(a) Awareness and knowledge of safe driving tactics (b) Awareness and knowledge of different types of hazardous material, markings, properties, and material safety data sheets (c) Awareness and knowledge of role in safety, security, and environment (d) Ability to perform risk assessment and hazard identification
Awareness and knowledge of **personal preparedness** as HAZMAT transportdriver	(a) Awareness and participation in HAZMAT disaster plan (b) Manage protective and medical aid equipment and decontaminate equipment as per emergency plan (c) Conduct contingencies regularly (d) Awareness of emergency services and their contact numbers
Awareness and knowledge on **actions required in an accident/incident** as HAZMAT transport driver	(a) Define warnings and reporting mechanism in case of HAZMAT accident (b) Knowledge of chain of command (c) Awareness and knowledge of the Incident Command System (d) Proficiency in use of fastest available communication means
**Knowledge of survival safety** steps	(a) Define health risks after exposure to HAZMAT (b) List different protective steps to mitigate the impact of HAZMAT agents (c) Knowledge of the use of personnel protective equipment (d) Knowledge of evacuation and cordoning
**Possess appropriate skills** required to address the incident	(a) Knowledge of first aid (b) Knowledge of triage in case of a mass causality incident (c) Awareness and knowledge of use of firefightingappliances (d) Knowledge of boiling liquid expanding vapor explosion (d) Awareness and knowledge of spill control techniques
Knowledge and **experience sharing** with others	(a) Participate openly in post-accident investigation and share actual occurrence (b) Maintain discipline and implement corrective preventive measures (c) Communicate about likely contamination of air, soil and water

## Discussion

Many departments and agencies have identified competencies required for first responders, however there are no identified competencies for HAZMAT transportation drivers.^32^–^36^ To the best of our knowledge; this is the first study of the competencies of HAZMAT transportation drivers dealing with emergency situations. In emergency situations, HAZMAT truck drivers are the first on-site to initiate emergency response measures. Although drivers may get on-the-job training, there is no program for educating drivers as first responders by incorporating these competencies into a professional educational course. The initial development program for HAZMAT truck drivers is the starting point; in developed nations, HAZMAT drivers are authorized to work only after obtaining proper driver training and approval. The same model can be considered for developing nations, with incorporation of the additional element of HAZMAT first responder training for drivers, as developing nations often do not have a robust emergency response system. The competencies proposed in this study could be used in development programs for drivers and cabin crews. Competencies were sorted based on emergency management phases. The concepts and sub-competencies can be used as reference material and guidelines for managers of organizations for implementation at different tiers.

The competencies identified here are based on previously acknowledged capabilities of first responders (i.e. emergency health professionals, fire fighters, HAZMAT response teams and law enforcement personnel) and were generated by experts with experience and knowledge of the subject. The competencies can be sorted into phases of emergency management: prevention, preparedness, mitigation, response, and recovery. The prevention actions focused on steps considered essential to prevent disasters, and the corresponding competencies involve proficiency in safe driving tactics and knowledge of HAZMAT properties. Drivers should be trained to conduct risk assessment and hazard identification. Identified measures to mitigate the effects of an emergency were awareness and knowledge of first aid, knowledge of triage and the ability to conduct triage in cases of mass causality incidents. To mitigate further loss in case of an accident, knowledge of boiling liquid expanding vapor explosions and spill control techniques are considered essential. Boiling liquid expanding vapor explosions are caused by the rupture of a container containing a pressurized liquid/liquefied gas when exposed to heat. This is directly related to the knowledge of HAZMAT types and properties. Awareness of and proficiency with firefighting techniques and equipment were identified as helpful in the mitigation phase.

Preparedness actions include awareness of emergency response plans, participation in emergency rehearsals, availability and knowledge of the proper use of personal protection equipment (PPE), medical equipment and spillage control equipment as outlined in an emergency response plan. The availability and use of appropriate PPE are key to an effective response.^37^ Face masks with designated respirators are also needed.^38^,^39^ Preparedness actions also include awareness and knowledge of partner emergency services such as 1 122 and other emergency contact numbers. Response actions include awareness of accident reporting mechanisms, ability to use the fastest available means of communication, liaise with emergency services and incident command, act promptly wearing PPE and controlling the spill or leakage, and ability to operate firefighting equipment if required. The final key competency is the ability to help in cordoning off the area to protect people and assets.

### Limitations

The first major limitation of the present study was that the research was conducted in a setting with limited industrial activities compared to more developed nations. Secondly, qualitative methods were used with a small sample size; a mixed method could yield more detailed data. The third major limitation of study is that it was conducted in the same geographic area; the diversity of Belt and Road Initiative project demands that further studies be conducted at different geographic locations to standardize the set of competencies. Finally, the identified competencies were not tested in real time emergency situations.

## Conclusions

The purpose of the present study was to explore and identify core and sub-competencies of HAZMAT truck drivers dealing with emergencies in Pakistan and other LMIC. As HAZMAT truck drivers are the first available at an incident site, they can swiftly initiate response actions. With training, it is possible to develop drivers that can act as first responders along with fire fighters, emergency medical nurses, HAZMAT teams and law enforcement personnel. At the policy level, HAZMAT drivers should be considered as first responders and thus development training programs for drivers and emergency responders should be similar. No previous study has considered the role of HAZMAT truck drivers as first responders. In developed nations, the emergency response mechanism is well developed and effective, but the initial response is critical. A HAZMAT driver trained as a first responder can initiate the required steps and play a vital role in emergency mitigation, especially in LMIC. A training program designed to train HAZMAT truck drivers as first responders should include the competencies identified in this study; initial training can be provided at driving training institutions, followed by on-the-job training and refresher courses. In addition, conducting regular exercises and drills is essential to ensuring effective responses. These practice drills are of more value if done in collaboration with other emergency services. The competencies identified can be utilized in different phases of disasters, i.e. prevention, preparedness, response, etc.

### Practical implications

The present study has a number of significant implications. First, oil and chemical transportation companies should consider the role of truck drivers as first responders and take appropriate measures to train drivers accordingly. Next, the selection, induction and training process of drivers should be improved, taking into account bodily, mental and psychological abilities to act as first responders. Salaries should include incentives for first responder training to provide additional motivation. Governments of underdeveloped/LMIC should revise their national transportation safety policies to designate HAZMAT drivers as first responders in case of accidents. Finally, hazardous material transport training programs should be established at the national level to impart standardized training to HAZMAT truck drivers.
